# Analysis of genotype resistance and HIV‐1 transmission risk in HIV‐1‐infected men who have sex with men in Guiyang, China

**DOI:** 10.1002/iid3.70029

**Published:** 2024-11-17

**Authors:** Dawen Qin, Zhangping Hong, Yi Wang, Nan Meng, Xueyu Yang, Du Shen, Yong Hu, Xinglin Yang

**Affiliations:** ^1^ Key Laboratory of Environmental Pollution Monitoring and Disease Control, Ministry of Education, School of Public Health Guizhou Medical University Guiyang China; ^2^ Department of Laboratory Guiyang Public Health Clinical Center Guiyang China; ^3^ School of Clinical Laboratory Science Guizhou Medical University Guiyang China

**Keywords:** gene locus mutations, genotype, HIV‐1 infected individuals, molecular transmission network, MSM

## Abstract

**Background:**

As the social economy has developed and population mobility has increased, differences in the Human immunodeficiency virus type 1 (HIV‐1) genotype distribution among men who have sex with men (MSM) have become apparent in the provinces and cities across China. The high variability and drug resistance characteristics of HIV‐1 can lead to the widespread spread of resistant strains, which may also result in antiretroviral therapy failure and an increase in the mortality rate.

**Objective:**

The genotypic drug resistance characteristics and HIV‐1 transmission risks among HIV‐1‐infected MSM in Guiyang, Guizhou Province were analyzed in the current study. The aim of the study was to provide a scientific basis for preventing the spread of HIV‐1 strains among MSM and develop intervention measures.

**Method:**

A cross‐sectional study was conducted at the Guiyang Public Health Clinical Center. A total of 181 HIV‐1‐infected MSM who not received treatment at the center between 1 January 2020 and 31 December 2022 were selected. The HIV‐1 *pol* region gene fragment, including the protease and reverse transcriptase regions, was amplified by nested PCR and RT‐PCR. The maximum likelihood method was used to construct a phylogenetic tree for analyzing the HIV‐1 genotypes in MSM. HIV‐1 genotypic resistance was evaluated using the Stanford University HIV drug resistance database. A molecular transmission network of HIV was constructed and the risk of HIV‐1 transmission was determined.

**Results:**

We successfully amplified 173 *pol* gene sequences from blood samples obtained from 181 patients. The main subtype was CRF07_BC (60.69% [105/173]), followed by CRF01_AE (26.59% [46/173]), CRF08_BC (4.05% [7/173]), CRF55_01B (4.62% [8/173]), B (3.47% [6/173]), and C (0.58% [1/173]). The distribution of HIV‐1 genotypes in MSM showed that there was a significant difference in the genotype composition of HIV‐1‐infected MSM according to registered residences and ages (*p* < .05). The CRF55_01B subtype accounted for the lowest proportion in Guiyang City and individuals >30 years of age. Multivariate logistic regression analysis of risk factors for drug resistance in HIV‐1‐infected MSM showed that the overall prevalence of pretreatment drug‐resistant species was 12.72% (22/173), and age >30 years, CRF55_01B subtype, and CD4^+^ T lymphocyte count >350 cells/mm^3^ were risk factors for drug resistance in MSM HIV‐1 strains. Among the pretreatment drug‐resistant species, non‐nucleoside reverse transcriptase inhibitors with ≥1 drug resistant‐species accounted for 9.25% (16/173), followed by protease inhibitors at 4.05% (7/173) and nucleoside reverse transcriptase inhibitors at 1.73% (3/173). Non‐nucleoside reverse transcriptase inhibitors resistant to the CRF07_BC and CRF01_AE genotypes were predominant. The CRF55_01B genotype was shown to be most likely to carry the V179E mutation. The molecular network included CRF07_BC and B genotypes. The results of multi‐factor logistic regression analysis on the factors affecting the rate of joining the network showed that individuals >30 years of age were less likely to join the network compared to those individuals <30 years of age.

**Conclusion:**

The distribution of HIV‐1 genotypes among MSM in Guiyang is diverse and complex. The main genotypes were shown to be CRF07_BC and CRF01_AE. The drug resistance mutation rate is high and pretreatment drug‐resistant species is at a moderate level of prevalence, with NNRTIs being the most common site for drug resistance mutations. The CRF07_BC subtype and patients <30 years of age were identified as the key intervention targets in Guiyang based on the molecular transmission network. Patients should routinely undergo drug resistance testing before starting antiretroviral therapy to avoid virologic treatment failure and prevent the spread of HIV‐1‐resistant strains in MSM.

## Graphical abstract

The distribution of HIV‐1 genotypes in MSM HIV‐1 infection is diverse and complex, and age is an important factor.

## 1 INTRODUCTION

The human immunodeficiency virus (HIV) is a retrovirus that mainly invades and destroys CD4^+^ T lymphocytes. The HIV RNA affects the immune system and functions of infected individuals as it replicates, and in severe cases can lead to various diseases (malignant tumors and opportunistic bacterial infections) that gradually develop into acquired immunodeficiency syndrome (AIDS).^1^ There were approximately 39 million people living with HIV worldwide by the end of 2022, two‐thirds (25.6 million) of whom were in the African region and approximately 630,000 HIV‐related deaths.^2^ China reported 1,260,928 HIV/AIDS patients and 437,307 deaths as of 30 June 2023.^3^

Due to the special high‐risk sexual behaviors of men who have sex with men (MSM), such as not using condoms and anal and oral sex, this group has become a high‐risk population for HIV‐1 infection. In China, the newly reported incidence of HIV infection in MSM increased from 9.1% in 2009% to 23.3% in 2018.^4^ Guiyang is the capital city of Guizhou Province and is adjacent to Yunnan, Sichuan, and Guangxi Provinces, where HIV‐1 infections are frequent. Due to the historical factors and unique geographic location, Guiyang has become an important hub for HIV‐1 transmission from border areas into mainland China. The prevention and control of HIV‐1 in MSM has become one of the important prevention and control priorities for AIDS in China.

When HIV‐1 patients are undergoing antiretroviral therapy (ART), drug‐resistant strains may develop due to factors such as pressure from drug selection. Pretreatment drug resistance (PDR) may include transmitted drug resistance and/or acquired drug resistance.^5^ However, in recent years there has been little data on the drug resistance characteristics of HIV‐1 genotypes among MSM in Guiyang. With the socioeconomic development and increased population flow in Guiyang, the distribution of HIV‐1 genotypes among MSM may become more diverse. Therefore, further research is needed to understand drug resistance characteristics.

In 2015 the United States used communication networks for analysis and proposed that intervention among MSM populations would reduce the risk of HIV infection in other high‐risk groups.^6^ Research on the molecular transmission network of HIV‐1 started later in China than in Western countries and is currently mainly used for HIV surveillance across various geographic regions. With the increasing prevalence of HIV‐1 drug‐resistant strains among MSM in Guiyang, it is essential to study the HIV‐1 genetic changes in this city to understand the transmission characteristics of drug‐resistant viruses. Therefore, we conducted a cross‐sectional study at the Guiyang Public Health Treatment Center and used molecular transmission networks to analyze the distribution of HIV‐1 genotypes and drug resistance characteristics among MSM. The aim of the study was to provide a reference for developing personalized clinical treatment plans for HIV‐1‐infected MSM as well as scientific evidence for disease prevention and intervention measures.

## 2 MATERIALS AND METHODS

### 2.1 Study design and participants

A cross‐sectional study was conducted at the Guiyang Public Health Clinical Center, which primarily treats HIV/AIDS patients from Guiyang City and surrounding cities (prefectures and counties) in Guizhou Province. The Center provides long‐term follow‐up care for patients and serves as a designated hospital for antiretroviral therapy. A total of 181 HIV‐1‐infected MSM who were treated at the Guiyang Public Health Clinical Center from 1 January 2020 to 31 December 2022 were selected for the current study. The inclusion criteria were as follows: (1) all patients were confirmed to be HIV‐1‐positive by the Guizhou Province HIV‐1 diagnosis laboratory based on western blot (WB) analysis; (2) HIV‐1 patients did not receive ART treatment before seeking medical attention; (3) the transmission route according to the patient questionnaire survey was MSM; (4) study participants have a household registration book or residence permit in Guizhou Province; (5) the HIV‐1 viral load was ≥1000 copies/mL; and (6) plasma samples were successfully collected from HIV‐1‐infected individuals and an informed consent form was signed. The exclusion criteria were as follows: (1) poor compliance; (2) mental illness; and (3) severe autoimmune disease. Basic epidemiologic data, such as ethnicity, registered residence, nature of household registration, age, educational level, marital status, reporting year, cigarette smoking status, and alcohol consumption status of the research subjects was recorded. This study was approved by the Medical Ethics Committee of Guiyang Public Health Clinical Center (approval number: 202148).

### 2.2 Laboratory testing indicators

EDTA anticoagulated peripheral venous blood was collected from HIV‐1‐infected MSM. The plasma was separated within 24 h after blood collection and stored in a −80°C freezer for future use. A BD FACSCanto^TM^ II flow cytometer (BD, USA) and a special reagent kit (BD) were used to determine the CD4^+^ T lymphocyte count. A Roche COBAS AmpliPrep automated instrument (Branchburg, NJ, USA) was used to determine the viral load.

### 2.3 HIV‐1 RNA extraction, nested PCR amplification, and sequencing

An HIV‐1 nucleic acid detection kit (Northeast Pharmaceutical Group Co., Ltd., Shenyang City, Liaoning Province, China) was used to extract viral RNA according to the manufacturer's viral RNA extraction protocol. We used nested polymerase chain reaction (PCR) to amplify the target gene fragment spanning the *pol* region (PR/RT region) of the reverse transcriptase and protease regions (HXB2: 2253 ~ 3312) with two rounds of PCR. A TaKaRa One‐Step RT‐PCR Kit (version 2, RR055A; TakaRa, China) was used for cDNA synthesis and the first round of PCR. A 25‐μL volume was used in the first round of the reaction system. The reaction procedure was as follows: reverse transcription at 50°C for 30 min; pre‐denaturation at 94°C for 2 min; 30 cycles of reaction (94°C for 30 s, 60°C for 30 s, and 72°C for 1 min); extension at 72°C for 10 min; and maintained at 4°C. The second round of RT‐PCR amplification was performed using the 2X Taq PCR Master Mix kit (Sangon Biotech Co., Ltd., Shanghai, China). A 50‐μL volume was used in the second round reaction system and the reaction procedure was as follows: pre‐denaturation at 94°C for 5 min; 30 cycles of reaction (94°C for 30 s, 63°C for 30 s, and 72°C for 2 min 30 s); extension at 72°C for 10 min; and maintained at 4°C. The PCR products from the second round were subjected to electrophoresis using a 1.5% agarose gel. The obtained target fragment samples were then sent to Sangon Biotech Co., Ltd. for purification and sequencing using the Sanger method. For relevant experimental operations, refer to the National Technical Specifications for AIDS Testing issued by the Chinese Center for Disease Control and Prevention.^7^ Amplification and sequencing primers are shown in Table 1.

**Table 1:** Nested PCR *pol* region gene fragment amplification and sequencing primers for MSM HIV‐1 infected individuals.

Procedure	Name	Sequences (5′‐3′)	Position
The first round RT‐PCR	RT21 (R)	CTGTATTTCTGCTATTAAGTCTTTTGATGGG	3539 ~ 3509
	MAW 26 (F)	TTGGAAATGTGGAAAGGAAGGAC	2028 ~ 2050
The second round PCR	RT20 (R)	CTGCCAGTTCTAGCTCTGCTTC	3462 ~ 3441
	PRO‐1 (F)	CAGAGCCAACAGCCCCACCA	2147 ~ 2166
Sequencing	RT20S3 (R)	GTTCTAGCTCTGCTTC	3456 ~ 3441
	RTB (F)	CCTAGTATAAACAATGAG ACAC	2946 ~ 2967
	PROC1S (R)	GCTGGGTGTGGTATTCC	2842 ~ 2826
	RTAS (F)	CTCAGATTGGTTGCAC	2524 ~ 2539
	PROS3 (F)	GCCAACAGCCCCACCA	2151 ~ 2166

Abbreviations: F, forward; R, Reverse.

### 2.4 Determination of HIV‐1 gene subtypes

The sequences obtained from sequencing were spliced using Sequencer 5.4 software and Bioedit 7.0 software was used for sequence comparison, manual correction, and cleaning. The length of the saved sequence was 1060 bp, which covered the protease region and part of the reverse transcriptase region (amino acids 1–99 and 1–254, corresponding to international reference strain HXB2: 2253 ~ 3312). A total of 27 international genotype reference strains, including A–C, F, H, J, K, and O, were downloaded from the Los Alamos HIV database (https://www.hiv.lanl.gov/content/index). The sorted sequences were compared with the reference strains using the MAFFT function in PhyloSuite software, and a maximum likelihood phylogenetic tree was constructed using the IQ‐TREE function of this database. Gene subtypes were determined based on bootstrap values >95%.

### 2.5 HIV‐1 genotype drug resistance analysis

According to the National Technical Specifications for AIDS Testing^7^ issued by the Chinese Center for Disease Control and Prevention, the sequenced data were submitted to the HIV Drug Resistance Database of Stanford University (https://hivdb.stanford.edu/). The HIVdb Program (Sequence Analysis online function [https://hivdb.stanford.edu/hivdb/by-sequences/]) of this database was used to evaluate the genotypic drug resistance mutation sites and drug resistance levels of HIV‐1‐infected MSM. The clinical relevance of drug resistance, including protease inhibitors (PIs), nucleoside reverse transcriptase inhibitors (NRTIs), and non‐nucleoside reverse transcriptase inhibitors (NNRTIs), is divided into five levels for each antiretroviral drug, as follows: susceptibility; potential low level of resistance; low level of resistance; moderate level of resistance; and high level of resistance. Low drug resistance and above were considered resistant.

### 2.6 Construction of molecular network for HIV‐1‐infected MSM

HyPhy2.2.4 software was used to analyze the good sequences and Tamura‐Nei 93 (TN93) model was applied to calculate the genetic distance between all sequences.^8^ The genetic distance results were screened using SAS 9.3 software and patients with a genetic distance ≤1.5% were considered to have a close relationship between infections, suggesting mutual transmission.^9^ Cytoscape 3.9.1 software was used to construct molecular networks and visualize the results.^10^

### 2.7 Statistical analysis

The database was constructed using Excel 2018. Statistical data analysis was performed using SPSS 24.0 (IBM Corp., Armonk, NY, USA). The normality of the data was assessed using the Kolmogorov–Smirnov test. The skewed distribution of quantitative data is described by the median {interquartile range [*M (P25, P75)*]}. Categorical variables are expressed as percentages and frequencies using the chi‐square test (χ^2^) or Fisher's exact probability method. Multivariate logistic regression analysis was used to analyze the risk factors of PDR and molecular network access rate. All tests were two‐tailed and a *p* < .05 was considered statistically significant. The number of mutation sites in the sample/the total number of samples successfully amplified by sequence × 100% = the detection rate of drug‐resistant mutation sites. When a sample contained multiple mutation sites, each different molecular site was counted separately for the calculation of the detection rate. The number of samples resistant to any drug/the total number of successfully amplified sequences × 100% = the detection rate of resistance.

## 3 RESULTS

### 3.1 Distribution of MSM HIV‐1 genotypes

**Figure 1:**
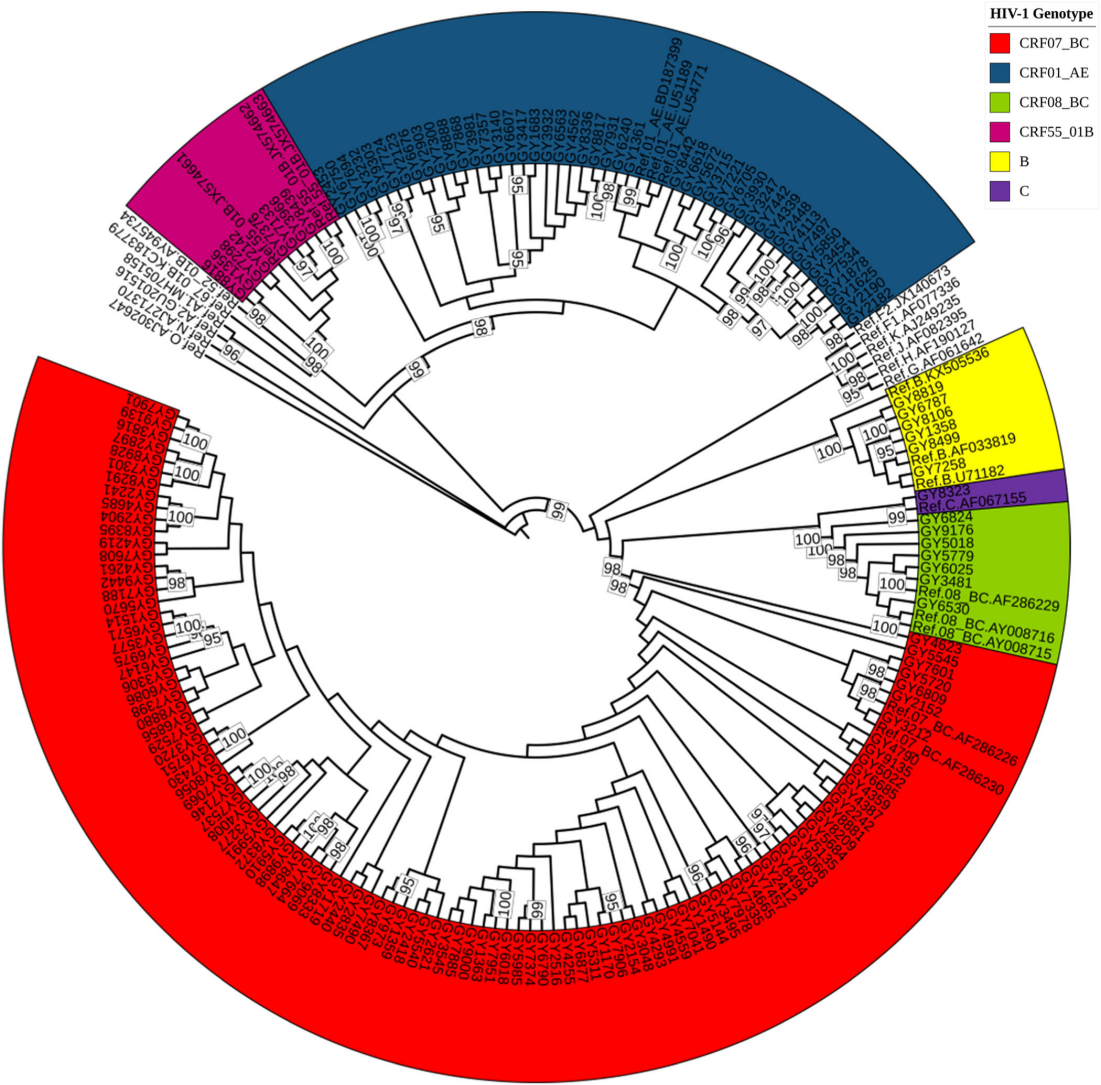
Maximum likelihood evolutionary tree of the HIV‐1 *pol* region sequence.

Among the 181 samples, 173 sequences were successfully amplified and there were 6 types of gene subtypes. CRF07_BC (60.69%, 105/173) was the main subtype, followed by CRF01_AE (26.59% [46/173]), CRF08_BC (4.05% [7/173]), CRF55_01B (4.62% [8/173]), B (3.47% [6/173]), and C (0.58% [1/173]). The two main MSM HIV‐1 subtypes accounted for 87.86% (151/173) of the overall subtypes (Figure 1).

### 3.2 Basic information of HIV‐1‐infected MSM

Of the 181 HIV‐1‐infected MSM, samples from 173 (95.58%) were successfully sequenced with ages ranging from 14 to 83 years and a median age of 29 years. The Han nationality accounted for 76.88% (133/173) of HIV‐infected MSM, registered residence of other regions in Guizhou Province accounted for 61.85% (107/173), and rural registered residence accounted for 52.60% (91/173). Of the respondents, 64.91% (95/173) had a college degree or above. The proportion of participants in the reporting year (2022) was relatively high (49.71% [86/173]). Of the participants, 54.91% (95/173) were non‐smokers and 51.45% (86/173) did not consume alcohol. The median (range) CD4^+^ T lymphocyte count was 266 (110–419) cells/mm^3^ and the proportion of patients with a CD4^+^ T lymphocyte count >350 cells/mm^3^ was 38.15% (66/173). The viral load range was mainly 1000‐100,000 copies/mL (67.63% [117/173]; Table 2).

**Table 2:** Demographic characteristics and genotype of MSM HIV‐1 infected individuals.

Variables	*N*	Subtype					
		CRF07_BC (*n* = 105)	CRF01_AE (*n* = 46)	CRF08_BC (*n* = 7)	CRF55_01B (*n* = 8)	Other^a^ (*n* = 6)	*P‐value* b
Ethnicity							0.183
Han Chinese	133	82(61.65)	37(27.82)	3(2.26)	5(3.76)	6(4.51)	
National minority	40	23(57.50)	9(22.50)	4(10.00)	3(7.50)	1(2.50)	
Registered residence							0.011
Guiyang City	66	40(60.61)	12(18.18)	5(7.58)	3(4.55)	6(9.09)	
Other regions in Guizhou Province	107	65(60.75)	34(31.78)	2(1.87)	5(4.67)	1(0.93)	
Nature of household registration							0.101
Countryside	91	55(60.44)	29(31.87)	2(2.20)	4(4.40)	1(1.10)	
City	82	50(60.98)	17(20.73)	5(6.10)	4(4.88)	6(7.32)	
Age (years)							0.005
≤30	109	68(62.39)	30(27.52)	0(0.00)	7(6.42)	4(3.67)	
>30	64	37(57.81)	16(25.00)	7(10.94)	1(1.56)	3(4.69)	
Education level							0.469
Junior high school and below	41	25(60.98)	12(29.27)	3(7.32)	0(0.00)	1(2.44)	
High school and technical secondary school	37	22(59.46)	9(24.32)	0(0.00)	3(8.11)	3(8.11)	
Junior college or above	95	58(61.05)	25(26.32)	4(4.21)	5(5.26)	3(3.16)	
Marital status							0.298
Married	24	14(58.33)	7(29.17)	3(12.50)	0(0.00)	0(0.00)	
Unmarried	138	83(60.14)	37(26.81)	3(2.17)	8(5.80)	7(5.07)	
Divorce/Widow	11	8(72.73)	2(18.18)	1(9.09)	0(0.00)	0(0.00)	
Confirmed year							0.081
2020	27	12(44.44)	11(40.74)	4(14.81)	0(0.00)	0(0.00)	
2021	60	39(65.00)	14(23.33)	1(1.67)	4(6.67)	2(3.33)	
2022	86	54(62.79)	21(24.42)	2(2.33)	4(4.65)	5(5.81)	
Smoke							0.781
Yes	78	49(62.82)	18(23.08)	4(5.13)	3(3.85)	4(5.13)	
No	95	56(58.95)	28(29.47)	3(3.16)	5(5.26)	3(3.16)	
Drink							0.906
Yes	84	49(58.33)	25(29.76)	3(3.57)	4(4.76)	3(3.57)	
No	89	56(62.92)	21(23.60)	4(4.49)	4(4.49)	4(4.49)	
CD4^+^T lymphocytes (cells/mm^3^)							0.765
≤200	59	38(64.41)	11(18.64)	3(5.08)	4(6.78)	3(5.08)	
201 ~ 350	48	30(62.50)	13(27.08)	1(2.08)	2(4.17)	2(4.17)	
>350	66	37(56.06)	22(33.33)	3(4.55)	2(3.03)	2(3.03)	
Virus load (copies/mL)							0.980
1000 ~ 100000	117	72(61.54)	30(25.64)	5(4.27)	5(4.27)	5(4.27)	
>100000	56	33(58.93)	16(28.57)	2(3.57)	3(5.36)	2(3.57)	

^a^ Other includes B subtype (N = 6) and C subtype (N = 1, pretreatment drug resistance = 1).

^b^ represents using Fisher's exact probability method.

### 3.3 Genotype distribution characteristics of HIV‐1‐infected MSM

The proportion of CRF07_BC was the highest in 2021 and 2022. There was no significant difference in the genotype composition of HIV‐1‐infected MSM with different ethnicities, household registration status, educational level, marital status, reporting year, cigarette smoking status, alcohol consumption habit, CD4^+^ T lymphocyte count, or viral load (*p* > .05). However, there were significant differences in the genotype composition of HIV‐1‐infected MSM with different registered residences and ages (*p* < .05). The CRF55_01B subtype accounted for the lowest proportion in Guiyang City, while other subtypes accounted for the lowest proportion in other regions of Guizhou Province. The CRF08_BC subtype was not detected in patients ≤30 years of age and the proportion of the CRF55_01B subtype was lowest among patients >30 years of age (Table 2).

### 3.4 Drug resistance of HIV‐1‐infected MSM

Of the 173 samples, 44 (25.43%) exhibited drug resistance site mutations. Because these mutations do not necessarily lead to drug resistance in HIV‐1 patients, it was determined that 22 cases were resistant, resulting in an overall drug resistance rate of 12.72% (22/173). There was no significant difference in the distribution of drug resistance among HIV‐1‐infected MSM with different ethnicities, registered residences, household registration status, educational level, marital status, reporting year, cigarette smoking status, alcohol consumption habit, or viral load (*p* > .05). However, the distribution of drug resistance in HIV‐1‐infected MSM varied significantly by age, genotype and CD4^+^ T lymphocyte count (*p* < .05). Individuals >30 years of age with the CRF55_01B subtype and a CD4^+^ T lymphocyte count >350 cells/mm^3^ were at risk for developing drug resistance to HIV‐1 strains (Table 3).

**Table 3:** Analysis of risk factors for drug resistance in MSM HIV‐1 infected individuals.

Variables	*N* (%)	PDR (%)	Crude OR (95% CI)	*P*‐value	Adjusted OR (95% CI)	*P*‐value
Ethnicity						
Han Chinese	133(76.88)	17(77.27)	1.000			
National minority	40(23.12)	5(22.73)	1.026(0.353‐2.98)	0.963		
Registered residence						
Guiyang City	66(38.15)	7(31.82)	1.000			
Other regions in Guizhou Province	107(61.85)	15(68.18)	0.728(0.280‐1.891)	0.514		
Nature of household registration						
Countryside	91(52.60)	10(45.45)	1.000			
City	82(47.40)	12(54.55)	0.720(0.293‐1.768)	0.474		
Age (years)						
≤30	109(63.01)	9(40.91)	1.000		1.000	
>30	64(36.99)	13(59.09)	0.353(0.142‐0.881)	0.026	0.315(0.122‐0.810)	0.017
Education level						
Junior high school and below	41(23.70)	5(22.73)	1.000			
High school and technical secondary school	37(21.39)	6(27.27)	0.718(0.199‐2.582)	0.611		
Junior college or above	95(54.91)	11(50.00)	1.061(0.344‐3.273)	0.918		
Marital status						
Married	138(79.77)	16(72.73)	1.000			
Unmarried	24(13.87)	4(18.18)	1.525(0.462‐5.029)	0.488		
Divorce/Widow	11(6.36)	2(9.09)	0.900(0.139‐5.844)	0.912		
Confirmed year						
2020	27(15.61)	2(9.09）	1.000			
2021	60(34.68)	8(36.36)	0.493(0.103‐2.357)	0.376		
2022	86(49.71)	12(54.55)	0.520(0.103‐2.631)	0.429		
Smoke						
Yes	78(45.09)	11(50.00)	1.000			
No	95(54.91)	11(50.00)	1.254(0.512‐3.069)	0.621		
Drink						
Yes	84(48.55)	9(40.91)	1.000			
No	89(51.45)	13(59.09)	0.702(0.283‐1.739)	0.444		
Genotype						
CRF07_BC	105(60.69)	12(54.55)	1.000		1.000	
CRF01_AE	46(26.59)	5(22.73)	1.058(0.350‐3.198)	0.920	0.707(0.216‐2.316)	0.567
CRF08_BC	7(4.05)	1(4.55)	0.774(0.086‐6.992)	0.820	0.550(0.052‐5.775)	0.618
CRF55_01B	8(4.62)	3(13.64)	0.215(0.046‐1.016)	0.052	11.254(1.714‐73.919)	0.012
Other^a^	7(4.05)	1(4.55)	0.774(0.086‐6.992)	0.820	1.212(0.112‐13.086)	0.874
CD4^+^T lymphocytes (cells/mm^3^)						
≤200	59(34.10)	3(5.08)	1.000		1.000	
201 ~ 350	48(27.75)	5(22.73)	0.307(0.461‐2.035)	0.307	0.394(0.087‐1.782)	0.227
>350	66(38.15)	14(63.64)	0.199(0.054‐0.732)	0.015	0.175(0.046‐0.660)	0.010
Virus load(copies/mL)						
1000 ~ 100000	117(67.63)	15(68.18)	1.000			
>100000	56(32.37)	7(31.82)	1.029(0.394‐2.688)	0.953		

^a^ Other includes B subtype (N = 6) and C subtype (N = 1, pretreatment drug resistance = 1).

MSM: men who have sex with men, DR: drug resistance, OR: odds ratio, CI: confidence interval.

### 3.5 Genotype drug resistance characteristics of HIV‐1‐infected MSM

**Figure 2:**
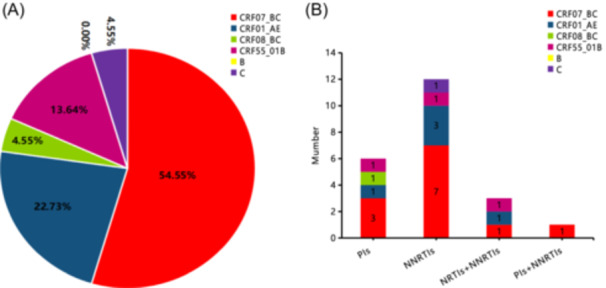
Analysis of resistance genotypes in the *pol* region gene sequence of MSM HIV‐1 infected individuals. (A) shows the proportion of drug‐resistant genotypes (22 cases of drug resistance). (B) shows the distribution of resistance genotypes among different drug types.

The proportions of genotypes among the 22 cases of drug‐resistant strains were as follows: CRF07_BC (54.55% [12/22]); CRF01_AE (22.73% [5/22]); CRF08_BC (4.55% [1/22]); CRF55_01B (13.64% [3/22]); and C (4.55% [1/22]). Most HIV‐1‐infected MSM (81.82% [18/22]) had PDR mutations that showed single drug resistance, while four strains (18.18% [4/22]) simultaneously carried two types of drug resistance. No three types of drug resistance were detected in the same individual. NNRTI resistance mainly involved the CRF07_BC and CRF01_AE genotypes, accounting for 72.73% (16/22) of all resistant strains. Among the overall HIV‐1‐infected MSM population, NNRTIs containing one or more drug resistance strains accounted for 9.25% (16/173), followed by PIs at 4.05% (7/173) and NRTIs at 1.73% (3/173; Figure 2).

### 3.6 Genotype drug resistance mutation sites in HIV‐1‐infected MSM

**Figure 3:**
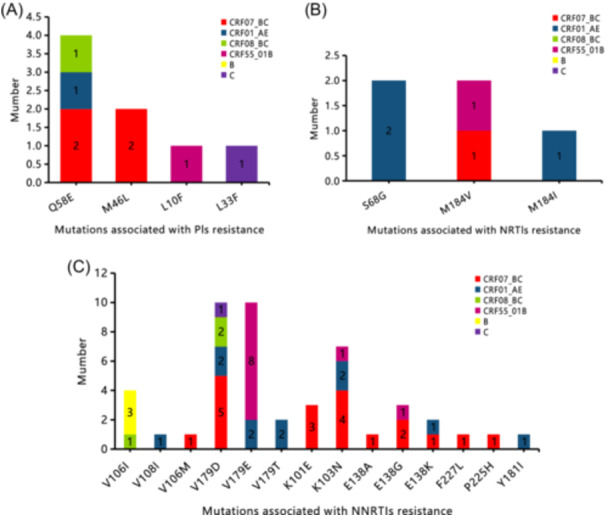
Analysis of resistance mutation sites in gene subtypes of MSM HIV‐1 infected individuals. (A) shows the mutation sites of genotype resistant PIs. (B) shows the mutation sites of genotype resistant NRTIs. (C) shows the mutation sites of genotype resistant NNRTIs.

Twenty‐one drug resistance mutation sites were identified, including four related to PI resistance, three related to NRTI resistance, and 14 related to NNRTI resistance. The overall PDR to NNRTIs (27.17% [47/173]) was much higher than NRTIs (2.89% [5/173]) and PIs (4.62% [8/173]) for HIV‐1‐infected MSM. The CRF07_BC subtype accounted for a relatively high proportion of mutations related to PIs. The main mutation sites included Q58E (2.32% [4/173]) and M46L (1.16% [2/173]). The NRTI‐related mutations were mainly the CRF01_AE subtype. The most common mutations were S68G (1.16% [2/173]) and M184V (1.16% [2/173]). Patients infected with CRF07_BC and CRF01_AE subtypes may develop NNTRI‐related mutations. The most common mutations were V179D (5.78% [10/173]), V179E (5.78% [10/173]), and K103N (4.05% [7/173]). The CRF55_01B genotype was most likely to carry V179E (Figure 3).

### 3.7 Drug resistance in HIV‐1‐infected MSM

Mutations associated with PIs led to fosamprenavir (0.58%), nelfinavir (1.73%), and tipranavir resistance (2.31%). Mutations associated with NRTIs conferred resistance to abacavir (1.73%), emtricitabine (1.73%), and lamivudine (1.73%). Mutations associated with NNRTIs conferred resistance to dolavirine (3.47%), efavirenz (8.09%), etravirine (2.89%), nevirapine (8.09%), and rilpivirine (5.20%). Most drugs showed potential low‐level resistance, while EFV and NVP were highly resistant mainly in the CRF07_BC subtype (Table 4).

**Table 4:** Analysis of resistance levels of different antiretroviral drugs in MSM HIV‐1 infected individuals.

Drug	Drug resistance level				
	*P* (%)	L (%)	I (%)	H(%)	PDR (%)
PIs					
ATV	2(1.16)	0(0.00)	0(0.00)	0(0.00)	0(0.00)
FPV	3(1.73)	1(0.58)	0(0.00)	0(0.00)	1(0.58)
IDV	3(1.73)	0(0.00)	0(0.00)	0(0.00)	0(0.00)
LPV	2(1.16)	0(0.00)	0(0.00)	0(0.00)	0(0.00)
NFV	5(2.89)	3(1.73)	0(0.00)	0(0.00)	3(1.73)
SQV	2(1.16)	0(0.00)	0(0.00)	0(0.00)	0(0.00)
TPV	3(1.73)	4(2.31)	0(0.00)	0(0.00)	4(2.31)
NRTIs					
ABC	0(0.00)	3(1.73)	0(0.00)	0(0.00)	3(1.73)
DDI	3(1.73)	0(0.00)	0(0.00)	0(0.00)	0(0.00)
FTC	0(0.00)	0(0.00)	0(0.00)	3(1.73)	3(1.73)
3TC	0(0.00)	0(0.00)	0(0.00)	3(1.73)	3(1.73)
NNRTIs					
DOR	4(2.31)	3(1.73)	2(1.16)	1(0.58)	6(3.47)
EFV	17(9.83)	3(1.73)	3(1.73)	8(4.62)	14(8.09)
ETR	26(15.03)	4(2.31)	0(0.00)	1(0.58)	5(2.89)
NVP	21(12.14)	2(1.16)	2(1.16)	10(5.78)	14(8.09)
RPV	22(12.72)	4(2.31)	1(0.58)	4(2.31)	9(5.20)

ATV (atazanavir), DRV (darunavir), FPV (fosamprenavir), IDV (indinavir), LPV (Kaletra), NFV (nelfinavir), SQV (saquinavir), TPV (tipranavir), ABC (abacavir), AZT (zidovudine), D4T (stavudine), DDI (didanosine), FTC (emtricitabine), 3TC (lamivudine), TDF (tenofovir), DOR (doravirine), EFV (efavirenz), ETR (etravirine), NVP (nevirapine), RPV (rilpivirine). P, potential resistance; L, low resistance; I, intermediate resistance; H, high resistance; L + I + H = Drug resistance.

### 3.8 Molecular transmission network in HIV‐1‐infected MSM

**Figure 4:**
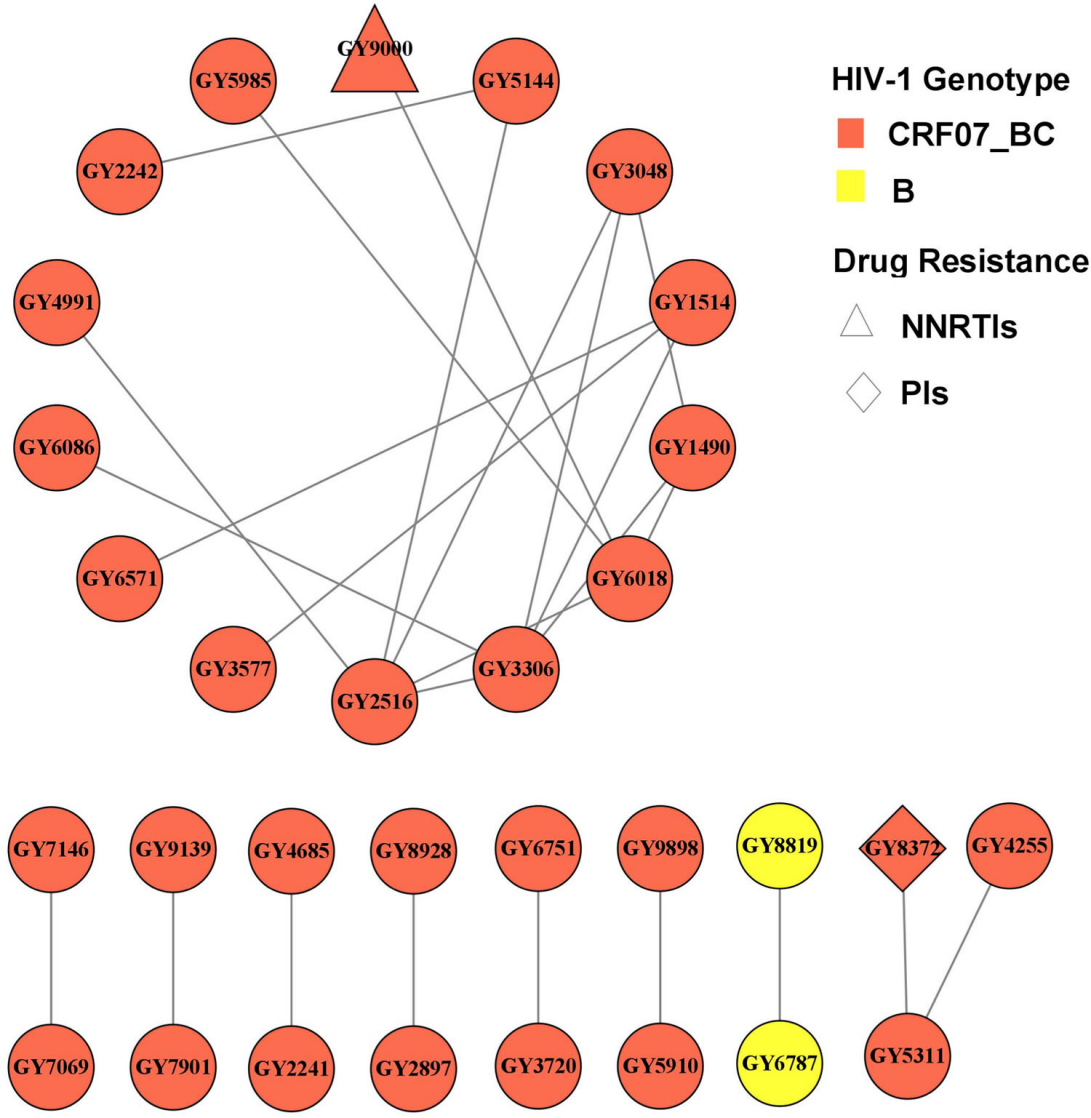
Molecular transmission network of *pol* region gene sequences in MSM HIV‐1 infected individuals. Setting the gene distance to be less than 0.015, 31 out of 173 gene sequences successfully entered the molecular propagation network.

A molecular network of HIV‐1 was constructed using 173 gene sequences from HIV‐1‐infected MSM. The genetic distance was set to <0.015, resulting in a molecular network consisting of 9 transmission clusters, 31 nodes, and 25 edges. Thirty‐one sequences entered the network with a net entry rate of 17.92% (31/173), among which the CRF07_BC subtype accounted for 93.55% (29/31) and the B subtype accounted for 6.45% (2/31). There were two cases (6.45% [2/31]) of CRF07_BC subtype resistance in the molecular network, including one patient who was resistant to NNRTIs with a mutation at K103N and showing high resistance to EFV and NVP. One patient was resistant to PIs with a mutation at position Q58E showing low‐level resistance to TPV (Figure 4).

### 3.9 Factors affecting the network access rate of HIV‐1‐infected MSM

The multi‐factor logistic regression analysis results of the factors influencing the patient network access rate showed that there was no significant difference in the network access rate between different ethnic groups, registered residences, household registration nature, educational level, marital status, reporting year, cigarette smoking status, alcohol consumption habit, CD4^+^T lymphocyte count, viral load, or drug resistance (*p* > .05). However, there was a significant difference between the network access rate and age (*p* < .05; Table 5).

**Table 5:** Factors influencing the internet access rate of MSM HIV‐1 infected individuals.

Variables	*N* (%)	Persons in TC, N (%)	Crude OR (95% CI)	*P*‐value	Adjusted OR (95% CI)	*P*‐value
Ethnicity						
Han Chinese	133(76.88)	21(15.79)	1.000			
National minority	40(23.12)	10(25.00)	1.778(0.757‐4.176)	0.187		
Registered residence						
Guiyang City	66(38.15)	14(21.21)	1.000			
Other regions in Guizhou Province	107(61.85)	17(15.89)	0.702(0.320‐1.539)	0.376		
Nature of household registration						
Countryside	91(52.60)	20(21.98)	1.000			
City	82(47.40)	11(13.41)	0.550(0.246‐1.231)	0.146		
Age (years)						
≤30	109(63.01)	25(22.94)	1.000		1.000	
>30	64(36.99)	6(9.38)	0.348(0.134‐0.900)	0.030	0.331(0.122‐0.895)	0.029
Education level						
Junior high school and below	41(23.70)	6(14.63)	1.000			
High school and technical secondary school	37(21.39)	5(13.51)	0.911(0.253‐3.278)	0.887		
Junior college or above	95(54.91)	20(21.05)	1.556(0.574‐4.214)	0.385		
Marital status						
Married	138(79.77)	25(18.12)	1.000			
Unmarried	24(13.87)	4(16.67)	0.904(0.284‐2.877)	0.864		
Divorce/Widow	11(6.36)	2(18.18)	1.004(0.204‐4.937)	0.996		
Confirmed year						
2020	27(15.61)	6(22.22)	1.000			
2021	60(34.68)	11(18.33)	0.786(0.257‐2.404)	0.673		
2022	86(49.71)	14(16.28)	0.681(0.233‐1.990)	0.482		
Smoke						
Yes	78(45.09)	16(20.51)	1.000			
No	95(54.91)	15(15.79)	0.727(0.334‐1.583)	0.421		
Drink						
Yes	84(48.55)	15(17.86)	1.000			
No	89(51.45)	16(17.98)	1.008(0.463‐2.194)	0.984		
Genotype						
CRF07_BC	105(60.69)	29(27.62)	1.000		1.000	
CRF01_AE	46(26.59)	0(0.00)	‐		‐	
CRF08_BC	7(4.05)	0(0.00)	‐		‐	
CRF55_01B	8(4.62)	0(0.00)	‐		‐	
Other^a^	7(4.05)	2(28.57)	1.048(0.193‐5.708)	0.957	1.139(0.199‐6.504)	0.884
CD4^+^T lymphocytes (cells/mm^3^)						
≤200	59(34.10)	8(13.56)	1.000			
201 ~ 350	48(27.75)	12(25.00)	2.125(0.789‐5.725)	0.136		
>350	66(38.15)	11(16.67)	1.275(0.475‐3.422)	0.630		
Virus load(copies/mL)						
1000 ~ 100000	117(67.63)	19(16.24)	1.000			
>100,000	56(32.37)	12(21.43)	1.407(0.629‐3.148)	0.406		
Drug resistance						
Yes	22(12.72)	2(9.09)	1.000			
No	151(87.28)	29(19.21)	2.377(0.526‐10.748)	0.261		

Abbreviations: CI, confidence interval; DR, drug resistance; MSM, men who have sex with men; OR, odds ratio; TC, transmission cluster.

^a^ Other includes B subtype (*N* = 6, TC = 2) and C subtype (*N* = 1).

## 4 DISCUSSION

A molecular epidemiologic study of HIV‐1‐infected MSM in Guizhou Province in 2011 identified three subtypes, with CRF07_BC accounting for the highest proportion (85.37%), followed by CRF01_AE (9.76%) and B (4.88%).^11^ The results of the phylogenetic tree constructed in this study showed that there were 6 HIV‐1 subtypes among HIV‐1‐infected MSM in Guiyang from 2020 to 2022, with CRF07_BC (60.69%) accounting for a higher proportion than CRF01_AE (26.59%), indicating an increase in multiple viral subtypes among HIV‐1‐infected MSM in Guizhou Province and maintaining predominance of the CRF07_BC subtype as the main epidemic strain. Therefore, strengthening the diagnosis and monitoring of the CRF07_BC subtype HIV‐1‐infected MSM population in Guizhou Province is recommended to control the prevalence of the CRF07_BC strain. The results of HIV‐1 genotype studies among MSM in Guiyang and some provinces in China are similar.^12^, ^13^ Since the first report of the CRF55_01B genotype in the MSM population in Shenzhen, China, the CRF55_01B genotype has been reported to be epidemic on a large scale in many places in China.^14^, ^15^, ^16^ This study showed that the prevalence of the HIV‐1 CRF55_01B subtype in MSM in Guiyang was 4.62%, mainly affecting unmarried MSM <30 years of age, suggesting that there may be cross‐provincial transmission of the CRF55_01B subtype strain. It is worth noting that most CRF55_01B strains carry the V179E mutation, which is more obvious than other mutations and has potential low‐level resistance to NNRTIs. Therefore, attention should be paid to the epidemiologic characteristics and clinical indicators of CRF55_01B strains, which will help to elucidate the reasons for its spread among MSM populations and prevent further spread.

The World Health Organization (WHO) has introduced the need to test for PDR in HIV‐1‐infected patients, which can help provide critical guidance on clinical medication treatment plans before and after exposure. The current study showed that 22 of 173 HIV‐1‐infected MSM in Guiyang had PDR resistance, with a high rate of drug resistance (12.72%), which is at the moderate epidemic level and close to the drug resistance rates (12.02%) in northern and southern areas of Guizhou Province in 2018,^17^ but higher than the 9.40% in Guiyang City in 2022.^18^ Multivariate logistic regression analysis showed that age >30 years, CRF55_01B subtype, and CD4^+^ T lymphocyte count >500 cells/mm^3^ were risk factors for HIV‐1 drug resistance in Guiyang MSM. This finding suggests that HIV‐1 drug‐resistant strains circulate frequently among this characteristic population and further monitoring of drug resistance should be strengthened in this group. The MSM population has the characteristics of strong concealment, multiple sexual partners, and diversity in sex behavior,^19^ which will accelerate the spread of drug‐resistant strains among the MSM population. The MSM population is also a potential “bridge” for sexually transmitted diseases to spread to the general population.

This study showed that NNRTIs accounted for 72.73% of all HIV‐1 drug‐resistant strains, with the CRF07_BC subtype resistance as the main type. This finding may be related to the lack of widespread use of PIs in Guiyang Province and may also be related to the high resistance barrier of PI‐based therapeutic drugs.^20^ This study showed that the CRF07_BC subtype accounted for a high proportion of single PI drug resistance mutations, mainly with Q58E and M46L mutation sites, which had low‐level resistance to TPV and NFV, respectively. Three cases were detected to have cross‐resistance to NRTIs and NNSTIs, with resistant drugs being ABC, FTC, and 3TC. NRTI drugs mainly have a mutation site at M184V/I, which can cause high resistance to FTC and 3TC. M184V/I at NNSTI drug‐related resistance mutation sites (K101E/K103N/E138K/F227L/V108I/V179E alleles) at the same time can lead to ABC drug resistance, which is similar to previous study results.^21^ This study showed that the CRF07_BC and CRF01_AE subtypes had a high incidence of NNTRI‐related mutations, the most common of which were V179D/E and K103N. The CRF07_BC and CRF01_AE subtypes exhibited resistance to EFV and NVP, which is consistent with related study findings.^22^ It has been reported that the V179D/E mutation site for the HIV‐1‐infected MSM CRF01_AE subtype has an increasing trend.^23^ It is noteworthy that this study showed that greater than one‐third (36.36% [16/44]) of the overall HIV‐1‐infected MSM drug resistance mutation sites (V179D/E) exhibit potential low‐level resistance to NNTRIs, which may lead to antiviral treatment failure in patients and requires further research.

The HIV molecular transmission network can effectively develop targeted interventions for high‐risk populations to improve public health efficiency.^24^ The results of this study on the construction of a molecular network for HIV‐1‐infected MSM showed that 31 sequences could be divided into nine transmission clusters, with the CRF07_BC subtype predominating. This finding suggests that the spread of the CRF07_BC subtype among HIV‐1‐infected MSM in Guiyang is severe. The results of the factors affecting infection in the network showed that age >30 years was a protective factor for entering the network, which may be related to the rapid growth of the Guiyang CRF07_BC subtype and the high proportion of patients with this genotype who were ≤30 years of age. In addition, there were two drug‐resistant patients ≤30 years of age in the molecular network who were resistant to NNRTIs and PIs. This finding suggests that drug‐resistant strains may have spread among the population. It is recommended to implement precise interventions and strengthen management measures for drug‐resistant patients to effectively control the further spread of drug‐resistant strains within active transmission clusters.

## 5 CONCLUSION

In summary, this study provided data on the epidemiologic characteristics and prevalence of PDR among HIV‐1‐infected MSM in Guiyang from 2020 to 2022. We have learned that the distribution characteristics of HIV‐1 genotypes among MSM in Guiyang are diverse and complex with CRF07_BC and CRF01_AE as the main genotypes. The drug resistance rate of HIV‐1‐infected MSM in Guiyang is high and at a moderate epidemic level with NNRTIs being the most common type of drug resistance. There is also cross‐resistance. Individuals >30 years of age with the CRF55_01B subtype and a CD4^+^ T lymphocyte count >500 cells/mm^3^ are risk factors for drug resistance among HIV‐1‐infected MSM in Guiyang. Drug resistance monitoring and evaluation should be further strengthened for this population. In addition, this study combined traditional epidemiologic data with molecular networks and found that age >30 years is a protective factor for network entry rate and the CRF07_BC subtype is the largest transmission cluster. Therefore, all HIV‐1‐infected MSM should be tested for genotype and PDR in a timely manner to better develop individualized treatment plans. This recommendation is of great significance in reducing the spread of drug‐resistant strains and accurately intervening in disease epidemics.

## AUTHOR CONTRIBUTIONS

Dawen Qin and Xinglin Yang conceived this study, and Yong Hu participated in the design and coordination. Dawen Qin, Zhangping Hong, Nan Meng, and Du Shen conducted HIV‐1 RNA extraction and nested PCR to obtain gene sequence data. Yi Wang, Xueyu Yang, and Xinglin Yang participated in the collection of epidemiological data. Dawen Qin analyzed the data and drafted a manuscript. Yong Hu and Xinglin Yang reviewed this manuscript. All authors have read and approved the final draft of the manuscript.

## CONFLICT OF INTEREST STATEMENT

The authors declare that there are no competing interests in this study.

## ETHICS STATEMENT

The research protocol was approved by the Medical Ethics Committee of Guiyang Public Health Clinical Center (approval number: 202148), and all subjects provided written informed consent.

## Data Availability

Under reasonable requirements, the data set analyzed in this study can be obtained from the corresponding authors. Upon reasonable request, the datasets analyzed in this study can be obtained from the corresponding authors.
